# RELoc: An Enhanced 3D WiFi Fingerprinting Indoor Localization Algorithm with RFECV Feature Selection

**DOI:** 10.3390/s26010326

**Published:** 2026-01-04

**Authors:** Shehu Lukman Ayinla, Azrina Abd Aziz, Micheal Drieberg, Misfa Susanto, Anis Laouiti

**Affiliations:** 1 Department of Electrical and Electronics Engineering, Universiti Teknologi PETRONAS, Seri Iskandar 32610, Malaysia; mdrieberg@utp.edu.my; 2 Department of Computer Engineering, University of Ilorin, Ilorin 240003, Nigeria; 3 Department of Electrical Engineering, University of Lampung, Bandar Lampung 35145, Indonesia; misfa@eng.unila.ac.id; 4 Samovar, Télécom SudParis, Institut Polytechnique de Paris, 9 Rue Charles Fourier, 91011 Evry, France; anis.laouiti@telecom-sudparis.eu

**Keywords:** extremely randomized trees (ERT), indoor localization, recursive feature elimination with cross-validation (RFECV), WiFi fingerprinting, Optuna-TPE

## Abstract

The use of Artificial Intelligence (AI) algorithms has enhanced WiFi fingerprinting-based indoor localization. However, most existing approaches are limited to 2D coordinate estimation, which leads to significant performance declines in multi-floor environments due to vertical ambiguity and inadequate spatial modeling. This limitation reduces reliability in real-world applications where accurate indoor localization is essential. This study proposes RELoc, a new 3D indoor localization framework that integrates Recursive Feature Elimination with Cross-Validation (RFECV) for optimal Access Point (AP) selection and Extremely Randomized Trees (ERT) for precise 2D and 3D coordinate regression. The ERT hyperparameters are optimized using Bayesian optimization with Optuna’s Tree-structured Parzen Estimator (TPE) to ensure robust, stable, and accurate localization. Extensive evaluation on the SODIndoorLoc and UTSIndoorLoc datasets demonstrates that RELoc delivers superior performance in both 2D and 3D indoor localization. Specifically, RELoc achieves Mean Absolute Errors (MAEs) of 1.84 m and 4.39 m for 2D coordinate prediction on SODIndoorLoc and UTSIndoorLoc, respectively. When floor information is incorporated, RELoc improves by 33.15% and 26.88% over the 2D version on these datasets. Furthermore, RELoc outperforms state-of-the-art methods by 7.52% over Graph Neural Network (GNN) and 12.77% over Deep Neural Network (DNN) on SODIndoorLoc and 40.22% over Extra Tree (ET) on UTSIndoorLoc, showing consistent improvements across various indoor environments. This enhancement emphasizes the critical role of 3D modeling in achieving robust and spatially discriminative indoor localization.

## 1. Introduction

The rapid evolution of the Internet of Things (IoT) and technological advancements has created a pressing demand for reliable and accurate location information [1]. This is crucial to propel the development of real-world applications, including intelligent urban infrastructure, industrial automation, emergency response, assistive navigation, robotics, and smart healthcare systems [2,3]. Outdoor localization has experienced substantial transformations, mainly due to advancements in satellite technology. On the contrary, indoor localization in satellite-compromised environments, including tunnels, campuses, transportation terminals, large shopping malls, and underground parking lots, poses significant challenges due to signal obstruction, which hinders Line-of-Sight (LoS) propagation between satellites and receivers [2]. Therefore, there is a need to provide precise location information to enhance the overall quality of life, boost productivity, and ensure the safety of personnel and assets. Researchers have explored different methods for indoor localization, with a WiFi fingerprinting approach emerging as a widely used solution [4,5,6,7,8]. This method offers a reliable, economical, and precise way to pinpoint locations within buildings or other confined areas. The practicality and affordability of WiFi-based fingerprinting methods offer excellent potential to transform indoor localization, instilling optimism about its future impact.

Indoor localization systems that utilize WiFi fingerprinting techniques, along with Artificial Intelligence (AI) algorithms such as Machine Learning (ML) and Deep Learning (DL), have emerged as robust solutions for several applications [6]. However, various detrimental factors significantly compromise their effectiveness, including temporal fluctuations in the WiFi received signal, multipath effects caused by obstacles, and human mobility within the area of interest [9]. Additionally, traditional 2D indoor localization approaches often fail in multi-floor environments due to vertical ambiguity (inter-floor confusion) and inadequate spatial representation. Thus, they do not offer the necessary precision required for most real-world applications [7,10,11]. These challenges emphasize the need for further research in this area, which this study aims to address. Integrating floor-level information in 3D coordinate prediction can offer more detailed information within buildings [12]. This integration provides a more realistic representation of the physical environment, enabling the differentiation of rooms located on different floors that may exhibit similar 2D Received Signal Strength Indicator (RSSI) patterns. This amalgamation is particularly crucial in advanced indoor localization applications where accurately identifying and distinguishing target locations is essential for high precision.

Traditional ML approaches often fail to accurately model the high dimensionality, nonlinearity, and noise inherent in WiFi RSSI data from complex indoor environments. While DL methods can capture these characteristics, they usually require extensive training time and significant computational resources. These challenges have limited their suitability for resource-constrained applications. To overcome these issues, Ensemble Learning (EL) has emerged as a promising alternative, providing a favourable balance between accuracy and efficiency [13]. These EL models efficiently process high-dimensional, nonlinear RSSI inputs while incurring less computational overhead than DL. In particular, Extremely Randomized Trees (ERT), an ensemble of randomized decision trees, offers strong generalization through diverse base learners and effective pattern recognition [14]. Empirical results in this work confirm that ERT attains competitive accuracy with significantly better computational efficiency than classical ML and DL baselines.

To the best of the author’s knowledge, no previous study has jointly addressed signal fluctuation and vertical ambiguity in multi-floor WiFi fingerprinting using a unified framework that combines Recursive Feature Elimination with Cross-Validation (RFECV) and ERT for 3D indoor localization. This paper introduces RELoc, which integrates RFECV for intelligent selection of the most discriminative APs and an Optuna TPE–optimized ERT regressor for 2D/3D coordinate prediction. The approach simultaneously enhances accuracy, reduces feature redundancy, and improves computational efficiency, addressing key limitations frequently overlooked in existing indoor localization pipelines. The core contributions of this study are summarized as follows:

▪ An RFECV-based feature selection pipeline that leverages cross-validation to identify the most spatially discriminative APs is introduced. This strategy significantly reduces input dimensionality while preserving informative feature sets.
▪ An enhanced WiFi fingerprinting framework called RELoc is proposed that achieves reliable and high-accuracy 2D and 3D coordinate regression.
▪ The effectiveness of the proposed RELoc is validated using the SODIndoorLoc and UTSIndoorLoc WiFi fingerprinting datasets, demonstrating strong generalizability and robustness across diverse indoor environments.
▪ The incorporation of floor-level information as an additional spatial dimension resolves inter-floor ambiguity and yields consistent performance gains up to 33.15% and 26.88% improvement over 2D localization on the respective datasets. This improvement highlights the necessity of vertical awareness in real-world indoor positioning systems.

The rest of the paper is organized as follows: In Section 2, related work is reviewed. Section 3 outlines a detailed methodology that covers the proposed feature selection, the ERT algorithm, hyperparameter optimization, dataset description, and performance metrics. Section 4 presents the findings and discusses them, while Section 5 concludes the study with recommendations for future research.

## 2. Related Work

This section discusses recent studies that have employed ML, EL, and DL algorithms for indoor localization. EL algorithms have consistently demonstrated superior performance over conventional techniques on typical tabular data [15]. These algorithms achieve high prediction accuracy without needing extensive data preprocessing such as filtering or normalization, thus minimizing training time and computational complexity.

Numerous approaches [16,17,18,19,20,21,22,23,24] have been proposed to enhance the performance of indoor localization systems. The study in [16] employs ML classification algorithms to assess the effectiveness of single and dual-band WiFi signals for indoor localization. The findings suggest that Adaboost and Artificial Neural Networks (ANN) yield the highest accuracy in both single-band (2.4 GHz) and dual-band (2.4 and 5 GHz). The authors in [17] propose a hybrid indoor positioning system that combines Principal Component Analysis (PCA), Weighted K-Nearest Neighbours (WKNN), and Linear Regression (LR) to enhance accuracy. This approach leverages PCA for dimension reduction, WKNN for handling noisy data and adapting to changing environments, and LR for location estimation. However, system complexity, reliance on high-quality data, and potential vulnerability to environmental changes are limitations that must be addressed.

In [18], an approach to indoor localization using dual-band WiFi was presented. The authors use a multi-view ensemble method to assess the performance of different algorithms, including KNN, XGBoost, Decision Tree, and Random Forest (RF). The study also utilizes eXplainable AI (XAI) frameworks to evaluate the effectiveness of single-band versus dual-band indoor localization systems and measure the contribution of each feature to localization accuracy. In [19], a supplementary open-source dataset, termed SODIndoorLoc, is introduced for research on WiFi indoor localization. This dataset contains multiple scenes in three distinct city buildings featuring corridors, offices, and meeting rooms. Experimental validation using various ML models yielded an average location accuracy of 2.3 m with RF. However, the studies in [18,19] are limited to 2D indoor localization.

The work in [20] presents an approach to indoor localization using Channel State Information (CSI) signals. The method involves fusing multi-view features and using AdaBoost to improve localization accuracy. However, the CSI signal requires additional hardware during measurements. A study in [21] introduces a technique known as XGBLoc for indoor localization. XGBLoc utilizes XGBoost and PCA to classify locations in multi-floor scenarios. This approach addresses the limitations of traditional indoor localization methods by leveraging XGBoost’s ability to handle large datasets and complex relationships. While the study achieved impressive accuracy, it is only constrained to 2D indoor localization.

The authors in [22] propose a method for enhancing indoor localization accuracy using ML classifiers. They collected RSSI samples from thirteen iBeacon nodes installed indoors. Their study compares the performance of different algorithms and recognizes KNN as the most effective with an accuracy of 85%. The article in [23] introduces a framework that leverages RSSI samples from the most robust AP and normalized output labels to enhance accuracy. The framework was tested using the UJI dataset, achieving 94.15% accuracy in floor classification and an average positioning error of 8.45 m using less than 5% of the 520 APs. A significant limitation of this study is its reliance on the availability and strength of WiFi signals, which can be susceptible to environmental factors. These factors include human absorption, building materials, and multipath effects, which can lead to signal variations and errors, ultimately impacting the overall accuracy. A recent study in [24] presents a framework to predict indoor floor/wing and 2D location coordinates. This approach uses a multi-channel Convolution Neural Network (CNN) that incorporates an attention mechanism referred to as MC-ACNNR. The results established by this approach are impressive, especially considering that no preprocessing is necessary. However, the complexity of the architecture may present considerable challenges for real-time applications on resource-constrained devices.

Recent studies have demonstrated notable advancements in indoor localization through the incorporation of data preprocessing pipelines, filtering techniques with sophisticated prediction algorithms, resulting in improved accuracy. However, these improvements often increase system complexity and resource requirements, which can limit their use in many environments. Moreover, conventional studies based on 2D indoor localization are only reliable in single-floor settings. However, they often fail in buildings with multiple floors due to inter-floor confusion and inadequate spatial representation. These challenges highlight the ongoing need for a robust and efficient framework to support seamless multi-floor navigation. The proposed method addresses this gap by incorporating floor-level information as an additional dimension to distinguish between locations on the different floors with similar RSSI patterns.

## 3. Materials and Methods

This section outlines the proposed method, including the feature selection employed, the EL used, the hyperparameter optimization strategy, the dataset utilized, and the evaluation metrics applied.

### 3.1. RFECV Feature Selection

Feature selection is crucial for developing efficient AI algorithms. It enhances their performance by refining the feature space. This process involves identifying and eliminating features that are irrelevant or contribute minimally to the algorithm’s predictive abilities. By employing feature selection techniques, models can reduce computational overhead, achieve higher accuracy, and improve interpretability [6]. Common metrics for evaluating the contribution of each feature to the predictive capability of algorithms include mutual information gain, feature variability, and importance scores. This study introduces RFECV, an intelligent feature selection technique that utilizes cross-validated scores to identify the optimal number of features. AP deployments in real-world environments are fixed and often redundant. This work focuses on optimizing input quality, rather than infrastructure design, by selecting the most spatially discriminative subset of existing APs to maximize indoor localization accuracy. The implementation pseudocode is presented in Algorithm 1.
**Algorithm 1**: Pseudocode for RFECV-based feature selection**Input**: **Training set**: (${Train}_{feature},{Train}_{target}$): ${Train}_{feature}\in R^{N\times d}$ → feature matrix, with N samples and d features and ${Train}_{target}\in R^{N\times 1}$ → corresponding target vector
${Base}_{Estimator}$: ERT_regressor
**CV parameters**: $K_{cv}\leftarrow 5$, Number of folds, Shuffle ← True
**RFE parameters**: Step size s←1, number of features to remove at each iteration, and $K_{min}\leftarrow 30$, min. number of features to retain
**Output**: Optimal feature set: $F^{*}\subseteq [f_{1},\;f_{2},\ldots ,\;f_{k}]$ and ${I(f}_{k}),$ importance score of each selected feature
  1.**Initialize** $F_{curr}\leftarrow {[f}_{1},\;f_{2},\ldots ,\;f_{d}]$, ${Best}_{score}\leftarrow 0$, and $F_{best}\leftarrow F_{curr}$ ∀ d>k  2.**While** $\left|F_{curr}\right|>K_{min}$ do
  a.**For** each fold ${K=1\;to\;K}_{cv}$ **do**
  i.Split ${Train}_{feature}\left[F_{curr}\right]$ and ${Train}_{target}$ into (${train}_{X},\;{train}_{y}$) and (${val}_{X},\;{val}_{y}$)
  ii.Fit ${Base}_{Estimator}$ on (${train}_{X},\;{train}_{y}$)
  iii.Predict on (${val}_{X},\;{val}_{y}$)
  iv.Compute validation ${score}_{K}$

      **end for**
     b.Compute the average CV score for the current feature set Avgscore=1Kcv∗sumK(scoreK)     c.**If** ${{Avg}_{score}>Best}_{score}$ then ${Best}_{score}={Avg}_{score}$ and $F_{best}=F_{curr}$     d.Compute feature importance score $I\left(f\right)$     e.Identify and remove the least importance features $F_{remove}\leftarrow [s\;features\;with\;lowest\;I(f)]$     f.Update the feature set $F_{curr}=F_{curr}-F_{remove}$
      **end if**
   **end while**
  3.**Return** $F^{*}\leftarrow F_{best}$ and ${I(f}_{k})$, the feature importance scores corresponding to $F^{*}$



### 3.2. Extremely Randomized Trees

An Extremely Randomized Trees (ERT) is an EL algorithm that combines the predictions of multiple randomized decision trees (base learners), similar to RF, but with added randomization. Unlike other ensemble models, it generates splits randomly without searching for optimal thresholds. Instead of bootstrap sampling, it typically uses the entire sample for each tree, maintaining randomization by selecting random split points [14]. This randomness fosters diversity among the trees and effectively balances the bias-variance trade-offs. Variance arises from the model’s excessive sensitivity to small fluctuations in the training sample, and high variance can lead to overfitting. This issue can be addressed by using explicit randomization in the selection of the feature subset and the choice of the cut point. Conversely, bias, which measures the ability to accurately generalize unseen data (with high bias potentially causing underfitting), is minimized by utilizing the entire original training sample to train each base learner [25]. These characteristics can lead to better generalization, and improved predictive performance.

The three fundamental parameters of the ERT model are the number of randomized decision trees in the ensemble $\left(N\right)$, the number of features to select randomly (K), and the minimum number of instances needed to split a node ($n_{min}$). Consider a training set with dimension $Q={[(r}_{1},l_{1}),\ldots ,{(r}_{j},l_{j}),\ldots ,{(r}_{z},l_{z})]$ with dimension (K+2), gathered from all nearby APs at z number of Reference Points (RPs) during the training phase, where $r_{j}\leftarrow (f_{1},f_{2},\ldots f_{K})$ is a set of K-dimensional RSSI fingerprints measured at j RPs and $l_{j}\leftarrow (x_{j},y_{j})$ is a 2D location coordinate at j RPs. The model generates N number of independent base learners. In each base learner, a subset $B_{p}$ of the training set Q is assigned to each child node p. Then, at each child node p, the model selects the optimal split based on the training subset $B_{p}$ and a random subset of the features using Algorithm 2. The subset $B_{p}$ at each child node p is divided into samples that satisfy the splitting rule, $B_{p}^{right}$ and the residual training samples, $B_{p}^{left}$. An MAE is employed as the scoring function to select the best split. The procedure continues at each child node p until it achieves the minimum required samples to split $n_{min}$, or when all the samples in the subset $B_{p}$ have the same label. Lastly, the label in the subset $B_{p}$ denotes the leaf node. During the test phase, a test set is passed to each base learner and across each child node, where the best splits guide the test set to the right or left child node until it reaches a leaf node. Then, the final prediction of the algorithm is calculated by averaging the predictions made by the N base learners.

**Algorithm 2**: Pseudocode for selection of ERT splitting rule
1. **Input**: training subset $B_{p}\leftarrow [b_{1},b_{2},\ldots ,\;b_{Y_{p}}]$     
K-dimensional vector from the sample $b_{k}\leftarrow (f_{1},\;f_{2},\ldots f_{K})$     
T← number of randomly selected features     
$n_{min\;}\leftarrow$ minimum number of instances needed to split a node
2. **If** $Y_{p}<n_{min\;}$ or all the node observations have identical label   
Stop splitting and identify the node as a leaf node
3. **else**   
Select a random subset of T features $\left(f_{1},\;f_{2},\ldots f_{T}\right)$ among original K features T≤K
4.   **For** each feature t in subset **Do**:     
Find $f_{t}^{max}$ and $f_{t}^{min}$ as max. and min. values of the feature t in subset $B_{p}$       Get a random cut-point, $f_{t}^{c}$, uniformly in the range $\left|f_{t}^{max}\;,f_{t}^{min}\right|$       Set $\left|f_{t}<f_{t}^{c}\right|$ as a potential split   
**end for**
5.   Choose a split $\left|f_{*}<f_{*}^{c}\right|$ so that $MAE{(f}_{*}^{c})={min}_{t=1,\ldots ,T}MAE{(f}_{t}^{c})$
6. **end if**
7. **Output**: **Return** the optimal split $\left|f_{*}<f_{*}^{c}\right|$ at the child node p. 

### 3.3. Optuna-Based Bayesian Hyperparameter Optimization

Generally, hyperparameter tuning is performed using manual, grid, or random search approaches. Grid search evaluates every potential combination of hyperparameter values defined in the parameter grid. In contrast, random search examines only a random subset of the possible hyperparameter combinations described in the predetermined parameter distribution. Selecting and evaluating candidate points without regard to previously assessed hyperparameters can lead to inefficiencies in both grid and random search methods, as considerable effort is wasted on evaluating redundant hyperparameters [26]. Thus, there is a growing demand for intelligent tuning methods that are efficient and achieve higher accuracy by considering the results of previously assessed hyperparameters.

Optuna-TPE is an automated hyperparameter optimization framework that employs TPE as the default algorithm. It efficiently navigates the search space to identify the optimal hyperparameter settings for an algorithm. Unlike traditional methods such as manual, grid, or random search, Optuna-TPE (a form of Bayesian optimization) uses a more intelligent strategy to select hyperparameter combinations that are more likely to improve performance. Instead of instinctively testing multiple hyperparameter combinations as in manual search, evaluating every possible hyperparameter combination as in grid search, or randomly selecting hyperparameter combinations as in random search, it employs a sampler to predict the most effective hyperparameter combinations. It focuses on promising regions of the search space and learns from previous attempts to narrow down to the best hyperparameter combinations swiftly [27]. This approach significantly enhances speed and efficiency. It relies on three fundamental optimization processes: an objective function, a trial, and a study. An objective function is a mathematical representation of the problem that Optuna aims to optimize, either by minimizing a loss (regression) or maximizing an accuracy (classification). It takes a trial argument as input and returns a validation score indicating performance. A trial is a single execution of the objective function with a specific set of hyperparameter combinations. A study refers to the entire optimization task, which involves multiple trials, and it aims to find the optimal solution.

For a training set $Q={\left[{(r}_{j},l_{j})\right]}_{j=1}^{N}$ where $r_{j}\in R^{d}$ is the RSSI measurements from multiple APs and $l_{j}\in R^{p}$ is the ground-truth location vector in a p-dimensional indoor space. During the optimization process, the aim is to learn a function $f_{x}$ so that $R^{d}\to R^{p}$ parameterized by the ERT hyperparameter x, that maps feature $r_{j}$ to the target locations $l_{j}$ with minimal error. Let $f_{x}(\cdot )$ denote the model, where x represents the hyperparameters, such as: $x_{1}$ → number of base learners (n_estimators), $x_{2}$ → a measure of the quality of a split (criterion), $x_{3}$ → maximum depth of each tree (max_depth), $x_{4}$ → number of features to consider when looking for the best split (max_features), $x_{5}$ → minimum samples to split a node (min_samples_split), and $x_{6}$ → minimum samples per leaf node (min_samples_leaf). The loss function ${L(f}_{x})$ is defined in Equation (1) with an MAE.

(1) $$L(x)=\frac{1}{N}\sum_{j=1}^{N}\left\|f_{x}\left(r_{j}\right)\;-\;l_{j}\right\|,$$

The goal is to identify the best set of hyperparameters $x^{*}$ as defined in Equation (2), where X is the feasible search space of the hyperparameters, x.

(2) $$x^{*}\;=\;{arg}_{x\in X}^{min}L(x),$$

Instead of modelling the L(x) directly as seen in Optuna-GP (Gaussian Process), TPE models the inverse conditional probability using two density functions as defined in Equation (3), where τ is a quantile (15%) of the lowest loss values observed so far, l(x) is the distribution of good hyperparameters and g(x) is the distribution of all other hyperparameters [28].

(3) $$p\left(x|L\left(x\right)\right)\propto \left\{\begin{array}{c} \;l(x),\;\;\mathrm{if}\;L(x)\;<\;\tau \\ g(x),\;\;\mathrm{otherwise} \end{array}\right.,$$

Optuna-TPE then selects the next candidate $x_{t+1}$ by maximizing the ratio in Equation (4).

(4) $$x_{t+1}={arg}_{x\in X}^{max}\frac{l(x)}{g(x)},$$

This approach favours sampling hyperparameters likely to belong to the region of good performance l(x). The l(x) and g(x) distributions are updated after each trial t, and the process continues until convergence is achieved or the maximum iteration is reached.

### 3.4. Dataset Description

**Figure 1:**
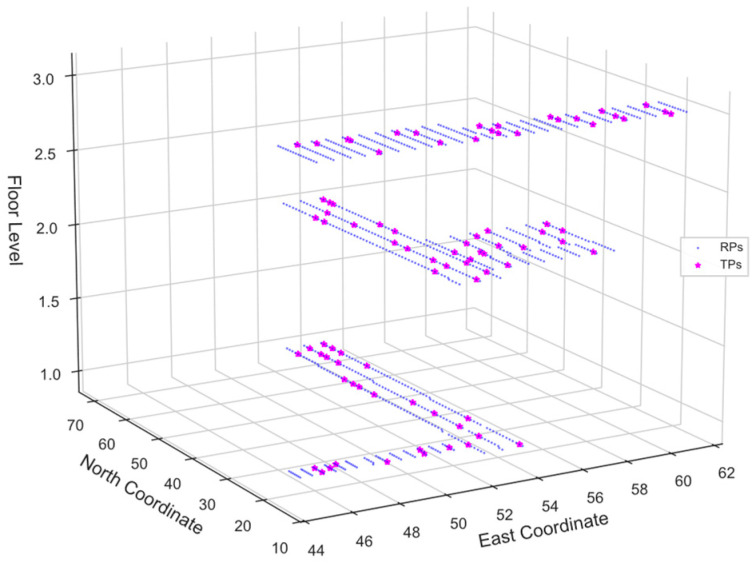
3D scatter plot showing the RSSI distribution at the RPs and TPs across the floors of the CETC331 building.

The effectiveness of the proposed RELoc is assessed using the SODIndoorLoc and UTSIndoorLoc datasets from diverse indoor environments. The SODIndoorLoc dataset compiled in 2022 has a total coverage area of about 8000 m^2^ [19]. It contains three buildings labeled CETC331, HCXY, and SYL, respectively. The dataset comprises 105 pre-installed APs across the buildings, with 56 single-band and 49 dual-band APs. The RPs are densely and uniformly distributed, with an average distance of about 1.2 m between adjacent RPs for the single-story buildings (HCXY and SYL) and 0.5 m for the three-story building (CETC331). The dataset comprises WiFi fingerprints collected at 1802 distinct points, consisting of 1630 RPs and 272 Testing Points (TPs). 23,925 RSSI samples were measured, with 21,205 (89%) dedicated to training and validation, and 2720 (11%) to testing. For this study, only the samples collected from the CETC331 building were used. Further details can be found in [19], where the dataset is published. Figure 1 visualizes the 3D scatter plot at the RPs and TPs where the training and test samples were gathered.

**Figure 2:**
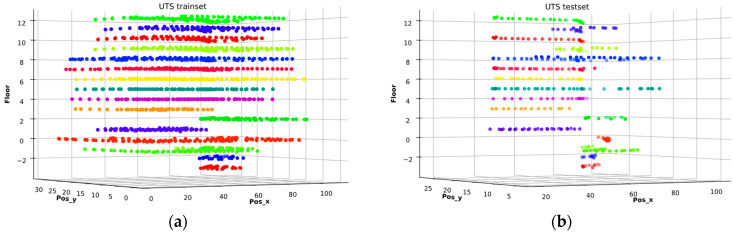
3D scatter plot showing the RSSI distribution at the (**a**) RPs and (**b**) TPs across the 16 floors of the UTS building.

The UTSIndoorLoc dataset was collected at the Faculty of Engineering and Information Sciences (FEIT) Building of the University of Technology Sydney (UTS) in 2019. It covers an area of about 44,000 m^2^ across 16 floors [29]. It includes 9496 samples, of which 9108 (96%) are designated for training and validation, and 388 (4%) for testing. Each sample comprises 589 RSSI features from various APs recorded from 1840 distinct points. The 3D scatter plot showing the distribution of RSSI values across x-y coordinates and floors within the building for training and test data is displayed in Figure 2, respectively.

### 3.5. Performance Metrics

ML algorithms are measured based on their performance on a dataset using various metrics. The selection of metrics depends on the problem being addressed. In this study, Mean Absolute Error (MAE), Root Mean Squared Error (RMSE), Coefficient of determination (R^2^), and Mean Localization Error (MLE) are employed as defined in Equations (5)–(8). In these equations, L denotes the number of test samples while A and E represent the actual and estimated X, Y, and Z coordinates, with their respective averages as $\bar{X}$, $\bar{Y}$, and $\bar{Z}$.

(5) $$MAE\;=\;\frac{1}{L}\sum_{1}^{L}\left(\left|X_{A}\;-\;X_{E}\right|\;+\;\left|Y_{A}\;-\;Y_{E}\right|\;+\;\left|Z_{A}\;-\;Z_{E}\right|\right),$$

(6) $$RMSE\;=\;\sqrt{\frac{1}{L}\sum_{i=1}^{L}{\left(X_{A}\;-\;X_{E}\right)}^{2}\;+\;{\left(Y_{A}\;-\;Y_{E}\right)}^{2}\;+\;{\left(Z_{A}\;-\;Z_{E}\right)}^{2}},$$

(7) $$R^{2}\;=\;1\;-\;\frac{\sum_{i=1}^{L}{\left(X_{A}\;-\;X_{E}\right)}^{2}\;+\;{\left(Y_{A}\;-\;Y_{E}\right)}^{2}\;+\;{\left(Z_{A}\;-\;Z_{E}\right)}^{2}}{\sum_{i=1}^{L}{\left(X_{A}\;-\;\bar{X}\right)}^{2}\;+\;{\left(Y_{A}\;-\;\bar{Y}\right)}^{2}\;+\;{\left(Z_{A}\;-\;\bar{Z}\right)}^{2}},$$

(8) $$MLE\;=\;\frac{1}{L}\sum_{i=1}^{L}\sqrt{{\left(X_{A}\;-\;X_{E}\right)}^{2}\;+\;{\left(Y_{A}\;-\;Y_{E}\right)}^{2}\;+\;{\left(Z_{A}\;-\;Z_{E}\right)}^{2}}.$$

## 4. Results and Discussion

This section presents the findings used to evaluate the performance of the proposed method. Simulations were conducted on a high-performance computing platform utilizing Python 3.11 with Jupyter (ipykernel 6.27.1), which runs on a 64-bit workstation featuring an Intel Core i3 processor clocked at 3.3 GHz and supported by 16 GB of RAM.

### 4.1. Hyperparameter Optimization Analysis

**Figure 3:**
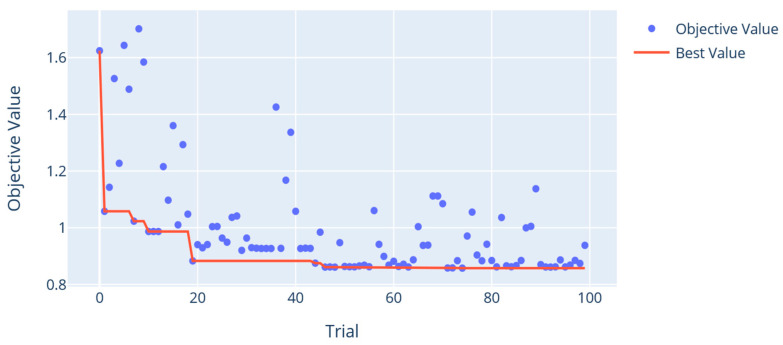
Optimization history plot of the ERT algorithm.

Figure 3 illustrates the optimization history over 100 independent trials. The first 40 trials display high variability, reflecting the TPE exploratory phase. After that, trials show convergence, with the lowest MAE of 0.86 m reached at trial 46. Further trials produce only insignificant improvements, indicating effective convergence and diminishing returns beyond this point. The overall trend in the optimization history confirms the algorithm’s effectiveness at efficiently identifying near-optimal hyperparameter settings.

**Figure 4:**
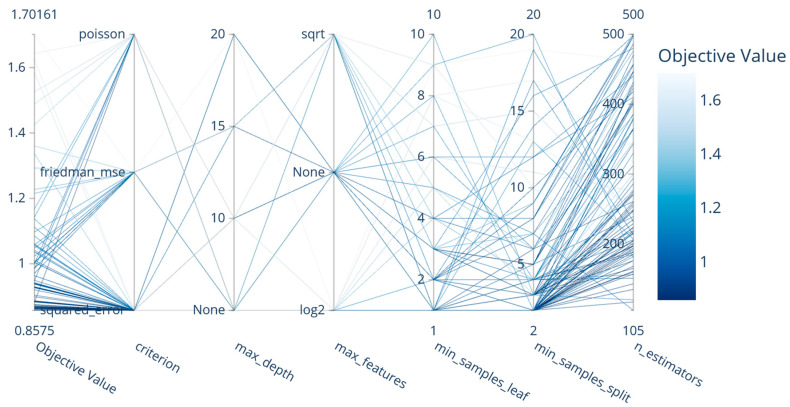
Parallel coordinate plot of the ERT algorithm.

The parallel coordinate plot in Figure 4 provides a detailed view of the hyperparameter space explored by the algorithm. The figure shows the hyperparameter setting that yields the lowest MAE, including 450 estimators, min_samples_split of 2, min_samples_leaf of 1, unlimited depth (max_depth = None), all features (max_features = None), and squared-error as the criterion. This setup indicates that unpruned ensembles with full feature access and minimal leaf constraints offer the best predictive accuracy in noisy RSSI environments, as they better capture complex, non-linear signal–location relationships.

**Figure 5:**
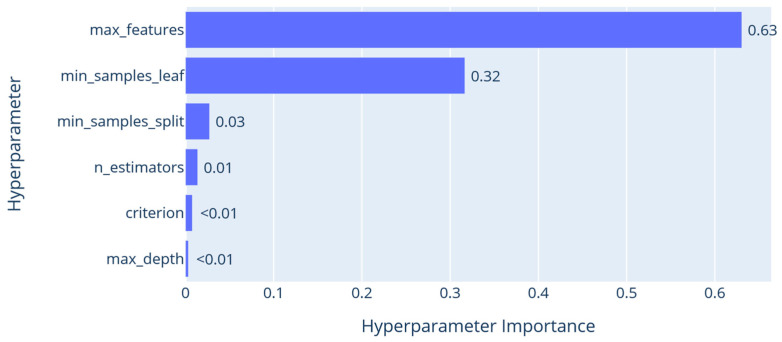
Hyperparameter importance plot of the ERT algorithm.

Figure 5 indicates hyperparameter importance, with max_features being the most significant (importance = 0.63). This suggests that allowing each split to consider all features, instead of a subset, is essential for effective discrimination in indoor localization. The min_samples_leaf (0.32) is the second most influential parameter, highlighting the advantage of detailed leaf resolution for capturing subtle spatial differences. In comparison, n_estimators (0.10), min_samples_split (0.03), and particularly criterion and max_depth (<0.01) have minimal impact. This indicates that once sufficient depth and leaf detail are established, increasing architectural complexity offers little additional benefit.

### 4.2. 3D Localization Performance on SODIndoorLoc

This subsection evaluates the performance of RELoc and other state-of-the-art methods, including Extra Tree (ET) [30], XGBoost (XGB) [21], Support Vector Machine (SVM) [31], Artificial Neural Network (ANN) [32], Deep Neural Network (DNN) [33], and Graph Neural Network (GNN) [34] on the SODIndoorLoc dataset for both 2D and 3D coordinate location prediction. All hyperparameters in Table 1 were determined via an automated process using Optun-TPE algorithm. The search space for each method was defined based on configurations reported in the relevant literature.

As shown in Table 2, the proposed 2D RELoc attains an MAE of 1.84 m, RMSE of 2.75 m, R^2^ of 90.44%, and MLE of 3.14 m. While a GNN baseline shows comparable 2D performance (MAE: 1.80 m, RMSE: 2.69 m, R^2^: 85.20%, MLE: 2.97 m), RELoc significantly outperforms other baselines (ET, SVM, XGB, ANN, DNN), with respective MAE improvements of 26.40%, 22.69%, 17.18%, 14.02%, and 8.91%. Consistent gains across RMSE, R^2^, and MLE further confirm RELoc’s regression fidelity.

**Figure 6:**
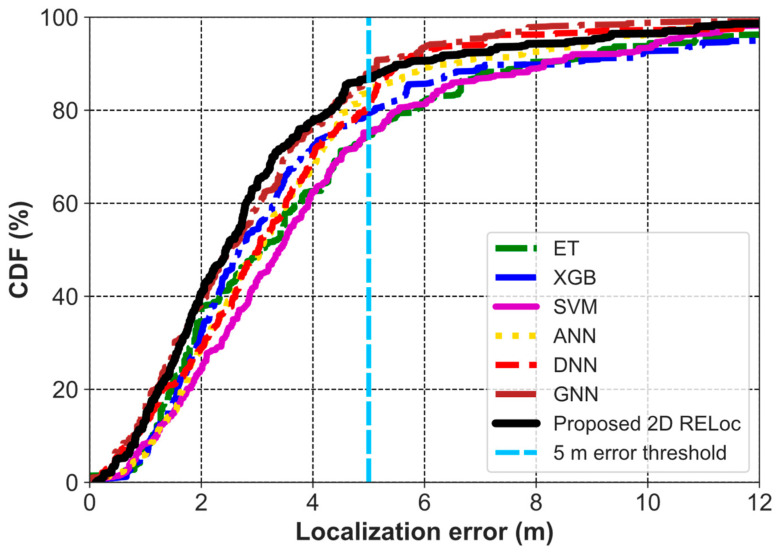
CDF of 2D Localization Error for RELoc and Baseline Methods on SODIndoorLoc.

The 2D localization error CDF in Figure 6 further validates this, showing that 87% of RELoc and GNN predictions lie within 5 m, surpassing ANN (83%), DNN (80%), XGB (79%), and SVM/ET (75%). The leftward shift in the RELoc and GNN curves indicates superior accuracy and robustness. Notably, RELoc achieves this performance with a less computationally intensive architecture than GNNs. However, in the tail of the error distribution (beyond ~5 m), the GNN and DNN exhibit slightly better performance than the proposed 2D RELoc, as shown in the CDF plot. This comparison highlights RELoc’s strong overall performance, particularly given the added complexity of the GNN approach.

**Figure 7:**
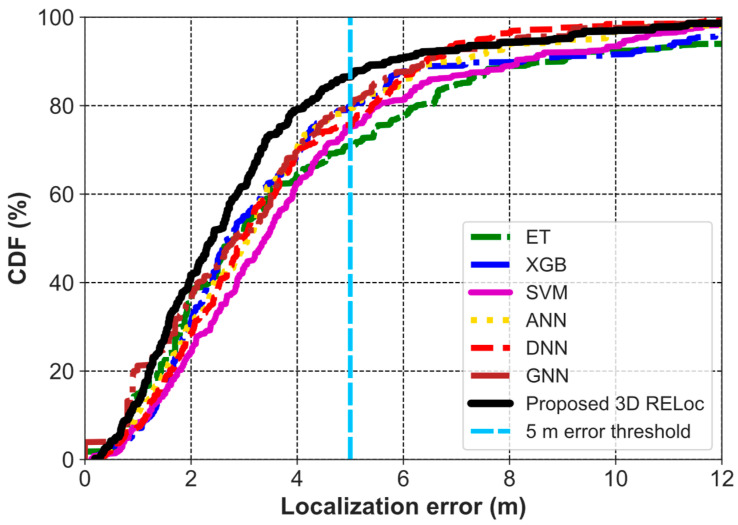
CDF of 3D Localization Error for RELoc and Baseline Methods on SODIndoorLoc.

Extending to 3D indoor localization, RELoc and the baselines incorporate floor-level information as an explicit spatial dimension, effectively resolving inter-floor ambiguity. The 3D RELoc achieves an MAE of 1.23 m, RMSE of 1.85 m, R^2^ of 93.25%, and MLE of 3.11 m, outperforming all baselines. This reflects 33.15% (MAE) and 32.73% (RMSE) improvements over its 2D counterpart. At a 5 m error threshold, as show in Figure 7, 3D RELoc achieves a success rate of about 90%, significantly outperforming GNN, ANN, and XGB (80%), DNN and SVM (75%), and ET (70%), demonstrating superior robustness and reliability in multi-floor environments. These improved results highlight that 3D modeling is essential for accurate, floor-aware indoor localization in multi-story environments.

### 4.3. 3D Localization Performance on UTSIndoorLoc

This subsection evaluates the performance of RELoc and other state-of-the-art methods on the UTSIndoorLoc dataset for 2D and 3D coordinate prediction to validate their robustness across different datasets. All baseline results in Table 2 and Table 3 were obtained by implementing the respective algorithms under identical experimental conditions as the proposed method.

**Figure 8:**
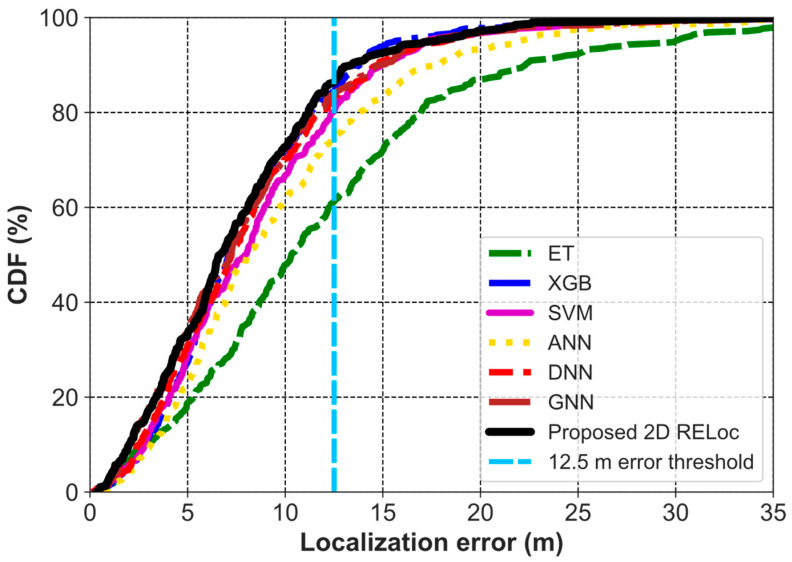
CDF of 2D Localization Error for RELoc and Baseline Methods on UTSIndoorLoc.

Among all the methods compared as presented in Table 3, the proposed 2D RELoc achieves an MAE of 4.39 m, an RMSE of 5.79 m, a R^2^ of 68.53%, and an MLE of 7.30 m. This is followed by XGB, GNN, and DNN, with similar performance but lower than the proposed method by about 10% (MAE). ANN has a slightly better performance compared to ET by 1.02 m, but lower than SVM by 1.02 m in terms of MAE. The CDF of localization error in Figure 8 indicates that the proposed 2D RELoc maintains consistency with 90% of the errors within a 12.5 m range.

**Figure 9:**
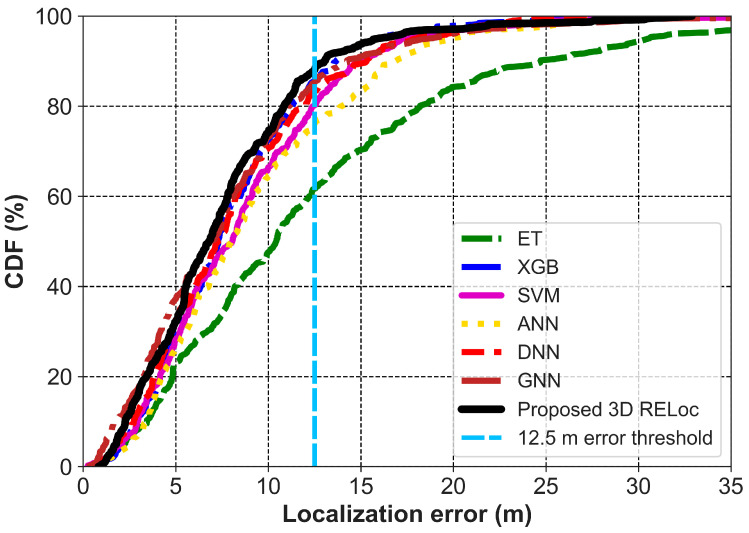
CDF of 3D Localization Error for RELoc and Baseline Methods on UTSIndoorLoc.

The proposed 3D RELoc reaches higher localization accuracy when floor-level information is included. Specifically, it achieves an MAE of 3.21 m, an RMSE of 4.16 m, an R^2^ of 78.41%, and an MLE of 7.60 m. These values correspond to the improvement of 26.88% in MAE, 28.15% in RMSE, and 12.60% in R^2^ compared to the proposed 2D method. However, a slight 3.95% increase in MLE is observed. Similarly to performance on SODIndoorLoc, RELoc outperforms the baseline methods, with an MAE improvement from 1.23% in GNN, 4.47% in XGB, 7.23% in DNN, 14.40% in SVM, 18.53% in ANN, to 40.22% in ET. As shown in Figure 9, the proposed 3D RELoc achieves 86% of predictions within 12.5 m, compared to 84% for GNN and XGB, 82% for DNN, 80% for SVM, 78% for ANN, and 61% for ET. This performance illustrates its superior robustness and reliability in multi-floor environments.

Table 4 presents comparative results for RELoc against the existing literature on the SODIndoorLoc and UTSIndoorLoc datasets. On SODIndoorLoc, 3D RELoc outperforms all baselines, achieving MAE improvements of 67.11% over RFR, 76.12% over MLPR, 49.38% over WKNN, 42.79% over 2D CNN-MS, 34.57% over VF-CLIP, and 40.29% over MC-ACNNR. On UTSIndoorLoc, although FALoc and MLP-SDAE report slightly lower MLE values than 3D RELoc, the proposed method achieves better RMSE, indicating enhanced overall coordinate estimation precision.

The experimental results across both SODIndoorLoc and UTSIndoorLoc consistently show that RELoc outperforms other benchmarked methods in both 2D and 3D coordinate prediction, achieving the lowest MAE, RMSE, and MLE while attaining the highest R^2^. This superior performance is primarily attributed to the intelligent feature selection mechanism (RFECV), which identifies and retains only the most spatially discriminative features, thereby enhancing model generalization and reducing noise-induced overfitting. Importantly, 3D RELoc resolves the persistent challenge of inter-floor ambiguity by incorporating floor-level information as an explicit spatial dimension. This enables robust differentiation of vertically aligned locations that exhibit near-identical 2D RSSI signatures, a common failure mode in conventional approaches. The framework’s ability to learn environment-specific RSSI variance further ensures reliable performance under real-world dynamics.

The CETC331 building in the SODIndoorLoc dataset has three floors with RPs densely and uniformly distributed across the three rooms on each floor, creating pronounced RSSI variations between the levels. This strong inter-floor signal differentiation enables RELoc’s 3D modeling to resolve vertical ambiguity, resulting in higher relative gains. UTSIndoorLoc, on the other hand, covers 16 floors of a single high-rise building with predominantly linear corridor layouts, as seen in Figure 2. The vertical signal decays slowly, and floor plans are very similar, leading to higher inter-floor RSSI similarity. As a result, while 3D modeling still enhances accuracy, the additional benefit is smaller compared to SODIndoorLoc.

Furthermore, SODIndoorLoc features 105 APs across 8000 m^2^ with 52 (26 dual bands) strategically placed on each floor in the CETC331 building to maximize spatial coverage. This results in a higher AP density per unit area, improving floor discriminability. UTSIndoorLoc, by contrast, deploys 589 RPs across 44,000 m^2^, with the larger footprint and vertical scale diluting AP density per floor. Moreover, the paper notes that the dataset includes basement levels and varied layouts [29], which can introduce further signal variability that slightly limits 3D gains. In addition, SODIndoorLoc utilizes sub-meter spacing (0.5 m in CETC331) to capture fine-grained RSSI gradients essential for 3D disambiguation. In contrast, UTSIndoorLoc has a coarser sampling density as indicated by 1,840 points over 16 floors, which reduces the resolution of vertical signal transitions.

**Figure 10:**
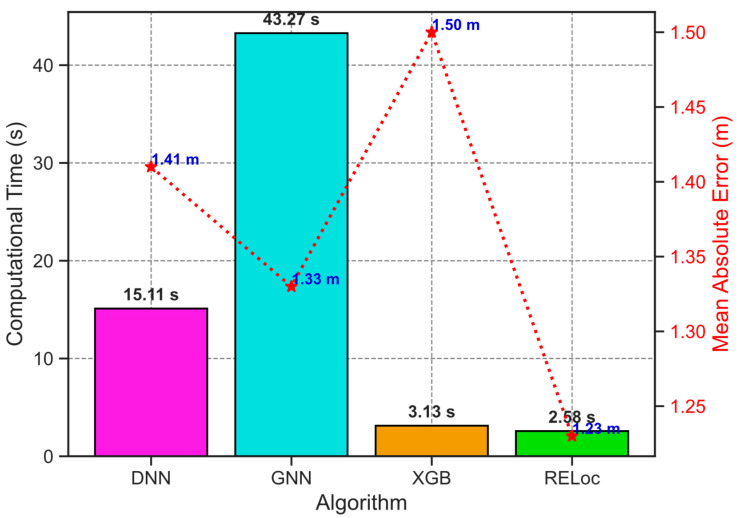
Computational time of RELoc, XGB, GNN, and DNN methods.

Beyond accuracy, RELoc exhibits notable computational efficiency. As shown in Figure 10, it achieves lower training latency than GNN by 40.69 s, DNN by 12.53 s, and XGB by 0.55 s while exceeding their localization accuracy, confirming its suitability for resource-constrained applications. Together, the integration of RFECV-based feature selection, 3D modeling, and an automated Bayesian optimization process via Optuna yields a solution that is not only accurate but also scalable, interpretable, and computationally efficient, addressing key barriers to real-world adoption of indoor localization systems.

## 5. Conclusions

This study presents RELoc, an accurate and efficient WiFi fingerprinting indoor localization method that combines RFECV for optimal feature selection and ERT for precise 2D and 3D coordinate prediction. The performance of RELoc was rigorously evaluated on SODIndoorLoc and UTSIndoorLoc datasets collected from multi-floor environments. The proposed 2D RELoc outperforms the state-of-the-art methods by achieving an MAE of 1.84 m and 4.39 m, on the respective datasets. Extending to 3D indoor localization, RELoc incorporates floor-level as an explicit spatial dimension, effectively resolving inter-floor ambiguity. On SODIndoorLoc, the 3D RELoc achieves an MAE of 1.23 m and an RMSE of 1.85 m, reflecting 33.15% (MAE) and 32.73% (RMSE) improvements over the 2D counterpart. The greater architectural heterogeneity, higher per-floor AP density, and finer spatial sampling in SODIndoorLoc enhance 3D modeling, resulting in the observed performance improvement. On UTSIndoorLoc, it reaches an MAE of 3.21 m and an RMSE of 4.16 m. These values correspond to the improvement of 26.88% in MAE and 28.15% in RMSE compared to the proposed 2D method. Furthermore, the performance of RELoc exceeds state-of-the-art methods, increasing from 7.52% to 40.11%. RELoc addresses inter-floor ambiguity and delivers better performance in multi-floor indoor localization by explicitly modeling vertical spatial structures and using intelligent feature engineering.

While this study employed the RFECV feature selection mechanism, future work can explore alternative techniques such as the Variance Inflation Factor (VIF), BorutaPy, Variance Threshold (VT), Mutual Information Gain (MIG), Relief Selector, or Minimum Redundancy Maximum Relevance (mRMR) to further enhance the effectiveness of feature selection. Additionally, the integration of eXplainable AI (XAI) frameworks, including Local Interpretable Model-agnostic Explanations (LIME), SHapley Additive exPlanations (SHAP), Explain Like I’m 5 (ELI5), and Descriptive mAchine Learning Explanations (DALEX), can be investigated to provide both local and global interpretability of the framework’s predictions. This integration will offer insights into the relative importance of selected APs and improving trustworthiness for real-world applications. Furthermore, beyond the TPE algorithm, future research can examine the impact of other Optuna-based optimization strategies, such as Random, Grid, Quasi-Monte Carlo (QMC), Non-dominated Sorting Genetic Algorithm III (NSGA-III), and Gaussian Process-based (GP) Bayesian optimization. This comparative study aims to provide a more comprehensive understanding of hyperparameter tuning strategies and their impact on model performance and generalizability in complex indoor scenarios.

## Figures and Tables

**Table 1:** Hyperparameter combinations for all the benchmarked methods.

Method	Hyperparameter Combination
ET	Criterion = squared error, splitter = random, max_features = 1.0, min_samples_split = 2, and min_samples_leaf = 1
XGB	n_estimators = 500, max_depth = 50, learning_rate = 0.01, objective = reg: squarederror, colsample_bytree = 0.1, min_child_weight = subsample = colsample_bylevel = 1.0
SVM	Kernel = RBF, C = 1, gamma = 0.01, epsilon = 0.001
ANN	HL = 256, 128, 64, max_iter = 100, learning_rate = constant, solver = Adam, activation = ReLU
DNN	HL = 256, 128, 64, learning_rate = 0.01, HL activation = ReLU, output activation = Sigmoid, dropout = 0.3, optimizer = Adam
GNN	Activation = ReLU, learning_rate = 0.001, dropout = 0.3, optimizer = Adam, Batch size = 8, epochs = 150

**Table 2:** 2D and 3D coordinate prediction of RELoc and other methods on SODIndoorLoc.

Coordinate	Method	MAE (m)	RMSE (m)	R^2^ (%)	MLE (m)
2D	ET	2.50	4.16	73.80	4.24
	XGB	2.22	3.23	88.21	3.83
	SVM	2.38	3.25	85.72	4.07
	ANN	2.14	2.95	87.55	3.60
	DNN	2.02	2.94	85.42	3.42
	GNN	1.80	2.69	85.20	2.97
	Proposed 2D RELoc	1.84	2.75	90.44	3.14
3D	ET	1.74	3.03	77.02	4.45
	XGB	1.50	2.20	91.59	3.86
	SVM	1.60	2.20	89.94	4.07
	ANN	1.44	2.04	91.56	3.63
	DNN	1.41	1.97	90.46	3.57
	GNN	1.33	1.98	88.19	3.36
	Proposed 3D RELoc	1.23	1.85	93.25	3.11

**Table 3:** 2D and 3D coordinate prediction of RELoc and other methods on UTSIndoorLoc.

Coordinate	Method	MAE (m)	RMSE (m)	R^2^ (%)	MLE (m)
2D	ET	7.33	9.62	2.23	11.92
	XGB	4.86	6.07	65.07	7.97
	SVM	5.29	6.77	52.19	8.55
	ANN	6.31	8.07	47.80	10.45
	DNN	4.95	6.29	63.05	8.14
	GNN	4.89	6.53	52.83	7.79
	Proposed 2D RELoc	4.39	5.79	68.53	7.30
3D	ET	5.37	7.41	23.09	12.41
	XGB	3.36	4.22	76.44	8.03
	SVM	3.75	4.84	66.24	8.63
	ANN	3.94	4.97	70.89	9.24
	DNN	3.46	4.50	73.59	8.16
	GNN	3.25	4.77	68.29	7.91
	Proposed 3D RELoc	3.21	4.16	78.41	7.60

**Table 4:** Performance comparison with existing approaches in the literature.

SODIndoorLoc				UTSIndoorLoc			
Ref.	Method	MAE (m)	RMSE (m)	Ref.	Method	RMSE (m)	MLE (m)
[19]	RFR	3.74	4.17	[35]	HDNN	−	7.80
[19]	MLPR	5.15	3.63	[36]	CAE-CNN	−	7.70
[37]	WKNN	2.43	−	[29]	CNNLoc	−	7.60
[38]	2D CNN-MS	2.15	−	[34]	GNN	−	7.48
[39]	VF-CLIP	1.88	2.53	[40]	MLP-SDAE	−	7.25
[24]	MC-ACNNR	2.06	3.08	[41]	FALoc	6.26	7.14
This work	2D RELoc	1.84	2.75	This work	2D RELoc	5.79	7.30
	3D RELoc	1.23	1.85		3D RELoc	4.16	7.60

## Data Availability

The datasets utilized are publicly available at [19,29].

## Abbreviations

The following abbreviations are used in this manuscript:

AI	Artificial Intelligence
ANN	Artificial Neural Network
APs	Access Points
CDF	Cumulative Distribution Function
CNN	Convolution Neural Network
CSI	Channel State Information
DL	Deep leaning
EL	Ensemble Learning
ERT	Extremely Randomized Trees
ET	Extra Tree
GPR	Gaussian Process
IoT	Internet of Things
MAC	Medium Access Control
MAE	Mean Absolute Error
ML	Machine Learning
MLE	Mean Localization Error
NLoS	Non-Line-of-Sight
RFECV	Recursive Feature Elimination with Cross Validation
RF	Random Forest
RMSE	Root Mean Squared Error
RPs	Reference Points
RSSI	Received Signal Strength Indicator
PCA	Principal Component Analysis
TPs	Testing Points
WiFi	Wireless Fidelity
WKNN	Weighted K-nearest Neighbours
XAI	eXplainable AI
XGB	eXtreme Gradient Boosting
